# IL-6 is increased in the cerebellum of autistic brain and alters neural cell adhesion, migration and synaptic formation

**DOI:** 10.1186/1742-2094-8-52

**Published:** 2011-05-19

**Authors:** Hongen Wei, Hua Zou, Ashfaq M Sheikh, Mazhar Malik, Carl Dobkin, W Ted Brown, Xiaohong Li

**Affiliations:** 1 Department of Neurochemistry, NY State Institute for Basic Research in Developmental Disabilities, New York, USA; 2 Department of Human Genetics, NY State Institute for Basic Research in Developmental Disabilities, New York, USA; 3 Shanghai Mental Health Center, Shanghai Jiao Tong University School of Medicine, Shanghai, China; 4 Department of Obstetrics & Gynecology, Nanfang Hospital, Guangzhou, China

**Keywords:** Autism, cytokines, synapse development, neural adhesion and migration, apoptosis, inflammation

## Abstract

**Background:**

Although the cellular mechanisms responsible for the pathogenesis of autism are not understood, a growing number of studies have suggested that localized inflammation of the central nervous system (CNS) may contribute to the development of autism. Recent evidence shows that IL-6 has a crucial role in the development and plasticity of CNS.

**Methods:**

Immunohistochemistry studies were employed to detect the IL-6 expression in the cerebellum of study subjects. *In vitro *adenoviral gene delivery approach was used to over-express IL-6 in cultured cerebellar granule cells. Cell adhesion and migration assays, DiI labeling, TO-PRO-3 staining and immunofluorescence were used to examine cell adhesion and migration, dendritic spine morphology, cell apoptosis and synaptic protein expression respectively.

**Results:**

In this study, we found that IL-6 was significantly increased in the cerebellum of autistic subjects. We investigated how IL-6 affects neural cell development and function by transfecting cultured mouse cerebellar granule cells with an IL-6 viral expression vector. We demonstrated that IL-6 over-expression in granule cells caused impairments in granule cell adhesion and migration but had little effect on the formation of dendritic spines or granule cell apoptosis. However, IL-6 over-expression stimulated the formation of granule cell excitatory synapses, without affecting inhibitory synapses.

**Conclusions:**

Our results provide further evidence that aberrant IL-6 may be associated with autism. In addition, our results suggest that the elevated IL-6 in the autistic brain could alter neural cell adhesion, migration and also cause an imbalance of excitatory and inhibitory circuits. Thus, increased IL-6 expression may be partially responsible for the pathogenesis of autism.

## Background

Autistic disorder is the most severe of a group of neurodevelopmental disorders, referred to as autism spectrum disorders (ASDs), and is characterized by problems in communication, social skills, and repetitive behavior. Susceptibility to autism is clearly attributable to genetic factors [1-4], but the etiology of the disorder is unknown. Recent studies suggest that a combination of environmental risk factors, autoimmune conditions and localized inflammation of the central nervous system may contribute to the pathogenesis of autism [5-10].

Interleukin (IL)-6 was originally found to be a major inducer of immune and inflammatory response [11]. Recent evidence points to a crucial role of IL-6 within the central nervous system (CNS). In the CNS IL-6 can trigger cellular responses mediating inflammation, neurogenesis, gliogenesis, cell growth, cell survival, myelination and demyelination [12-16]. IL-6 is normally expressed at relatively low levels in the brain [15]. However, in the presence of brain injury or inflammation, IL-6 is elevated in the cerebral spinal fluid and brain homogenates [13,14]. Chronic over-expression of IL-6 in transgenic mice causes neuroanatomical and neurophysiological alterations associated with neurological disease [14,17]. IL-6 and leukaemia inhibitory factor were found to promote astrocytic differentiation of neural stem⁄progenitor cells [18]. Most recently, Oh *et al *demonstrated that IL-6 promotes specific neuronal differentiation of neural progenitor cells from the adult hippocampus [19].

Recent studies have reported an association of cytokines with autism. TNF-α, IFN-γ, IL-1β and IL-12 were found to be elevated in blood mononuclear cells, serum and plasma from autistic subjects [6,20-24]. Employing a cytokine PCR array, Vargas *et al *[7] demonstrated that IL-6, TNFα, transforming growth factor (TGF)-β1 and macrophage chemo-attractant protein (MCP)-1 were increased in autistic brains. In addition, MCP-1, IL-8 and other proinflammatory molecules were also found to be significantly elevated in the cerebrospinal fluid of autistic children [7]. Consistent with the these findings, our studies using a multiple bead immunoassay showed that IL-6, TNFα, IL-8, GM-CSF and IFNγ were significantly increased in the fontal cortices of autistic subjects as compared with the age-matched controls [5]. All these findings suggest that the immune system and cytokines may play important roles in the pathogenesis of autism. However, the mechanisms by which immune dysfunction and cytokine alteration contribute to the pathogenesis of autism remain unknown. In this study, we examined IL-6 in the cerebellum of autistic subjects. Our results showed that IL-6 was significantly increased in the cerebellum of autistic subjects as compared to age-matched controls. In addition, we over-expressed IL-6 in cerebral granule utilizing a viral construct expression approach to study the effects of IL-6 on neural cell properties and synapse formation. We further demonstrated that IL-6 over-expression in granule cells caused impairment in adhesion and migration. IL-6 had little effect on the formation of dendritic spines or on granule cell apoptosis. However IL-6 over-expression stimulated the formation of granule cell excitatory synapses, while having no effect on inhibitory synapses. Our results provide further evidence for an association of aberrant IL-6 expression with autism. Impaired neural cell adhesion and migration, as well as the excessive formation of excitatory synapses caused by elevated IL-6 expression could be an underlying cellular mechanism partially responsible for the pathogenesis of autism.

## Methods

### Study subjects

Frozen human brain tissues of six autistic subjects (mean age 8.3 ± 3.8 years) and six age-matched normal subjects (mean age 8 ± 3.7 years) were obtained from the NICHD Brain and Tissue Bank for Developmental Disorders (Baltimore, MD21201). Donors with autism fit the diagnostic criteria of the Diagnostic and Statistical Manual-IV, as confirmed by the Autism Diagnostic Interview-Revised. Samples were excluded from the study if they had a diagnosis of fragile X syndrome, epileptic seizures, obsessive-compulsive disorder, affective disorders, or any additional psychiatric or neurological diagnoses. The subject information is summarized in Additional file 1 Table S1.

### Immunohistochemistry

Paraffin sections, 6 μm thick, from 10% formalin-fixed cerebellar specimens from both autistic subjects and control subjects were deparaffinized with Xylene and ethanol and then washed with Tris [tris(hydroxymethyl)aminomethane]-buffered saline (TBS). The sections were then incubated with primary antibodies (IL-6, 1:100, Abcam) overnight at 4°C. After washing in TBS for 5 min, the sections were further incubated with the secondary antibody (biotinylated horse anti-rabbit IgG, VectaStain Elite ABC Kit, Vector Lab) for 30 min at RT, followed by incubation in Avidin-biotinylated peroxidase (VectaStain Elite ABC Kit) for 45 min at RT and in 0.0125 g DAB/25 ml 0.05 M TBS/1 drop 30% H2O2 for 10 min at RT. All sections were dehydrated with ethanol and Xylene before mounting for examination by confocal microscopy (Nikon Eclipse 90i, 10 × 40 magnification). Image J analysis (National Institutes of Health, Bethesda, MD, USA) was used to calculate areas and immunostaining densities.

### Cell culture

Cerebellar granule cells (CGCs) were prepared from C57BL/6J mouse pups (5-6 days postnatal) as described previously [25]. Briefly, the entire cerebellum was dissected out, and single cell suspensions were prepared by trypsinization and trituration. Cells were seeded into PDL-coated dishes. After 24 h, the medium was replaced with serum-free medium containing 15% N-2 supplement (Invitrogen) and 15 mM KCl.

### *In vitro *adenoviral gene delivery

Mouse IL-6 GFP adenovirus (IL-6 group) and GFP adenovirus (Control group) were generated by Welgen (Worcester, MA). After culturing for 4-5 days, CGCs were infected with Ad-GFP-IL-6 or Ad-GFP at multiplicity of infection (MOI) of 500. After 24 h, the medium was replaced with normal growth medium. Three days after infection, CGCs were used for the experiments reported in the present study.

### Cell adhesion assay

10, 000 CGCs were plated per well in 96 well tissue culture plates coated with recombinant ICAM-1 (inter-cellular adhesion molecule 1, R&D Systems) at the final concentration of 10 μg/ml. After 1.5 h of attachment, unattached cells were removed by aspiration and adherent cells were quantitated by the colorimetric aqueous MTS assay (CellTiter 96 AQ_ueous _One Solution kit, Promega).

### Cell migration assay

CGCs were labeled with fluorescent Calcein AM (BD Biosciences) at a final concentration of 2.5 μM. 10, 000 labeled cells were plated in 0.8 ml DMEM +1% FBS in each well of 24 well chambers adapted for the HTS Fluoroblock (BD Falcon) apparatus. After 3 h, 5% FBS was added to the lower chamber medium to establish a 1-5% serum gradient. Migration of cells from the upper to lower chamber were quantitated at 2 h using a microfluorimetric plate reader (CytoFluor 4000, MTX Lab Systems).

### DiI labelling

The cultured cells were labeled using a protocol adapted from Hering *et al *[26]. Briefly, CGCs were fixed in 4% formaldehyde for 15 min and incubated with Vybrant-DiI cell-labeling solution (1:200, Invitrogen) for 25 min at 37°C. Cultures were washed in warmed PBS, incubated in PBS at 4°C for 24-48 h to allow dye diffusion within membranes, mounted on glass slides with ProLong Gold antifade reagent (Invitrogen), and then imaged using Nikon Eclipse E800 microscope. Mature dendritic spines appear mushroom or stubby-shaped morphology; while the immature spines appear thin morphology [27,28]. For quantification of the density of dendritic spines, a dendritic protrusion with an expanded head that was 50% wider than its neck was defined as a spine [29]. The number of spines from one neuron was counted manually and normalized per 50 μm dendritic length.

### TO-PRO-3 Staining

To exam the apoptotic effect of IL-6 on CGCs, TO-PRO-3 staining was used to visualize the nuclear morphology of apoptotic cell. Cells that had bright condensed, fragmented nuclei after staining were considered to be apoptotic [30,31]. To identify cells as neurons, the culture was stained with anti-MAP2 polyclonal IgG (1:50, Cell Signaling Technology) and Alexa Fluor 555-conjugated anti-rabbit IgG (Invitrogen). After incubation with the secondary antibody, the CGCs were stained with TO-PRO-3 iodide (final 1 mM; Invitrogen) for 15 min, the cells were mounted and observed for nuclear changes under a Nikon eclipse 90i confocal laser scanning microscope. The maximal wavelength of excitation/emission of the TO-PRO-3 dye is 642/661. Lasers with excitation wavelengths of 633 nm were applied to activate the far-red fluorescence which was converted to blue under the laser confocal system. The experiment was repeated for three times.

### Immunofluorescence

CGCs were fixed in 4% formaldehyde for 15 min and blocked with 3% goat serum/0.3% Triton X-100 in PBS and incubated with anti-Syp polyclonal antibody (Synaptophysin, 1:200, Cell Signaling Technology), anti-VGLUT1 monoclonal antibody (1:500, Millipore), and anti-VGAT polyclonal antibody (1:500, Millipore) overnight at 4°C, followed by incubation with Alexa Fluor 577 anti-mouse and anti-rabbit IgG (1:1000, Invitrogen) for 1.5 h at room temperature. Cultures were mounted on glass slides with ProLong Gold antifade reagent (Invitrogen), and then imaged using Nikon eclipse 90i confocal laser scanning microscope.

The analysis of immunofluorescence images were done as described previously [32]. Images were acquired in the linear range with constant settings and analyzed using ImageJ software. All analyses were performed blind to the treatment of the culture. Immunoreactive puncta were defined as discrete regions along the dendrite with fluorescence intensity twice the background and average size of the puncta were normalized with data from Ad-GFP group, respectively. For quantification, 20-30 neurons from two to three different batches of cultures and experiments for each condition were randomly chosen on the basis of healthy morphology. Negative controls, in which the primary antibodies were omitted and treated only with the secondary antibodies, were run for each condition to exclude false positive secondary antibody binding. The n value refers to the number of cells analyzed.

### Statistics

We determined the statistical significance among groups with the unpaired t test using the StatView 5.0 software (SAS Institute, Inc.). A null hypothesis probability of <0.05% was considered significant. All data are presented as means ± standard error (SEM).

## Results

### IL-6 expression was increased in the cerebellum of autistic subjects

**Figure 1:**
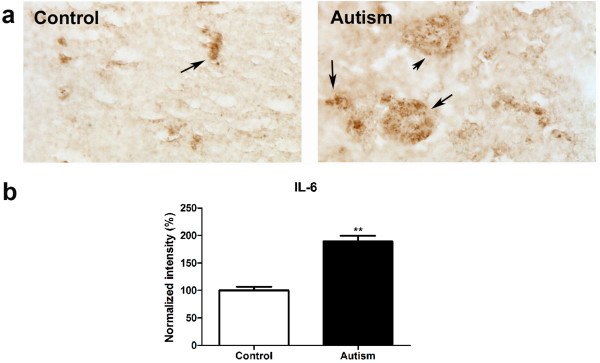
**IL-6 expression increased in the cerebellum of autistic subjects**. Immunohistochemistry studies were carried out on cerebellar homogenates from 6 autistic subjects and 6 age-matched controls using an IL-6 antibody (dilution 1:100). Stronger immunostaining of IL-6 (dark brown color indicated by an arrow) was present in the autistic samples (a). Immunostaining density was quantified using Image J analysis (b). Data are shown as mean ± SEM.

Immunohistochemistry studies were conducted to examine IL-6 expression in the cerebellum of six autistic subjects and six age matched controls using IL-6 antibody. The results are shown in Figure 1. We observed that the positive staining of IL-6 is obviously stronger in the cerebellum of autistic subjects as compared with age-matched controls. Quantitative analysis using Image J showed that IL-6 is significantly increased by 89% in autistic samples (p < 0.01).

### *In vitro *adenoviral gene delivery

**Figure 2:**
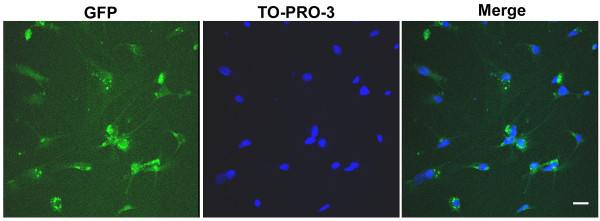
***In vitro *adenoviral gene delivery**. The GFP expression on the CGCs shown after 24 h infection. The nuclei were labeled by TO-PRO-3 dye. Scale bar, 25 μm.

The *in vitro *experiments confirmed CGCs were efficiently infected by an adenoviral vector carrying the GFP. The infection efficiency as revealed by GFP signal was over than 90% (Figure 2).

### IL-6 over-expression inhibited CGCs adhesion

**Figure 3:**
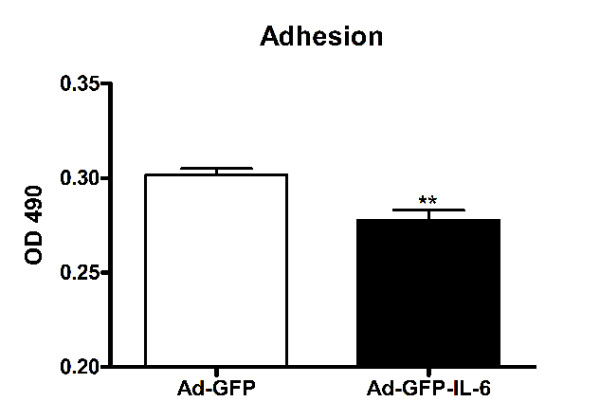
**IL-6 over-expression inhibited CGCs adhesion**. Comparision of CGCs adhesion between the control group and IL-6 group. The values are mean ± SEM from three independent experiments. OD: optical density. **P < 0.01 compared with control condition.

To test the effect of IL-6 on adhesion of CGCs, CGCs transfected with GFP tagged IL-6 viral vector (Ad-GFP-IL-6) or GFP viral vector alone (Ad-GFP, used as a control) were seeded on the ICAM-1-coated surface. The efficiency of cell adhesion was evaluated by a modified MTS assay based on the dehydrogenase conversion of MTS to colored tetrazolium salt, which occurs only in viable cells that adhered to the substrate. The amount of colored product formed at 490 nm (OD 490) was proportional to the number of attached cells. As shown in Figure 3, IL-6 inhibited cell adhesion by approximately 8% (p < 0.01, Figure 3).

### IL-6 over-expression impaired granule cell migration

**Figure 4:**
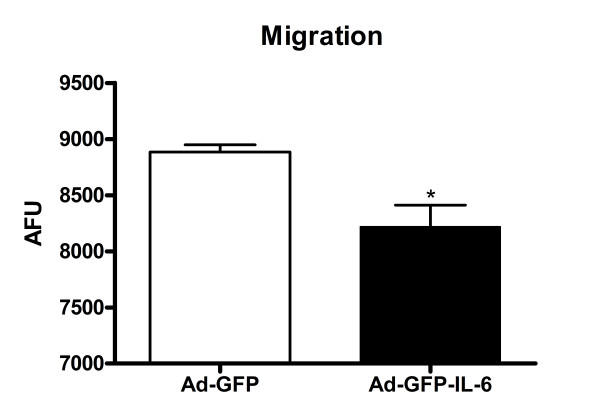
**IL-6 over-expression impaired granule cell migration**: Modified Boyden chamber migration assays on IL-6 and control CGCs. The number of migrated cells was determined by fluorescence readings. The values are mean ± SEM from three independent experiments. AFU: arbitrary fluorescence units. *P < 0.05 compared with control condition.

Cell migration is modulated by a complex of adhesion molecules between migrating cells and surrounding extracellular matrix (ECM) proteins, and critically affected by cell adhesion [33]. Since we observed decreased adhesion in IL-6 over-expressing cells, we investigated whether IL-6 plays a role in CGC migration with a modified Boyden chamber assay [34]. Our results showed that the migration of IL-6-infected CGCs, like cell adhesion, was also decreased by approximately 8%, as compared with the controls (p = 0.01, Figure 4).

### IL-6 had no detectable effect on the formation of dentritic spines

**Figure 5:**
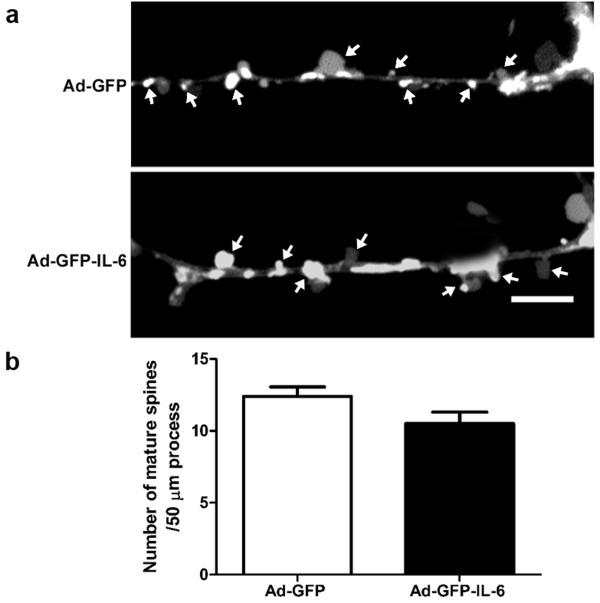
**IL-6 had little effect on the formation of dentritic spine**. (a) Cultured CGCss were infected with IL-6 or control vector, and then stained with Vybrant-DiI to outline the shape of dendritic spines. Arrowheads show the mature spines of mushroom-type. (b) The histogram shows the density of mature spines per 50 μm dendrite in CGCs transfected with IL-6 (n = 10 neurons) or control vector (n = 8 neurons). The data are the mean ± SEM. Scale bars, 5 μm.

To test whether over-expression of IL-6 affected dendritic spine generation, DiI labelling was employed to show their density. Control CGCs showed spines at a density of 12.40 ± 0.65 spines per 50 μm dendrite length, while IL-6 CGCs showed a density of 10.50 ± 0.80 (p = 0.08, Figure 5). These results indicate that IL-6 over-expression had no detectable effect on the formation of dendritic spines.

### IL-6 over-expression did not promote granule cell apoptosis

**Figure 6:**
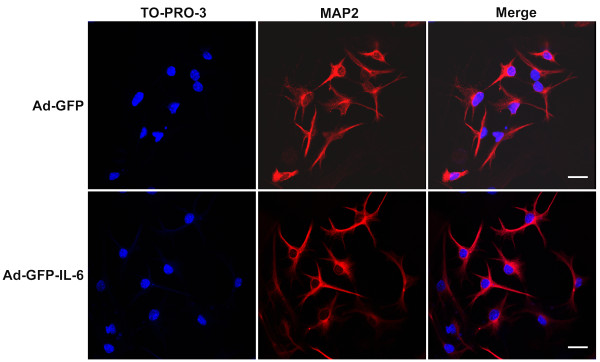
**IL-6 over-expression did not promote granule cell apoptosis**. Representative images of labeling of TO-PRO-3 (blue) to show the morphology of nuclear and MAP2 (red) in IL-6 and control CGCs, respectively. No typical apoptotic CGCs were observed in either control or IL-6 group. Scale bar, 25 μm.

Granule cell apoptosis can be induced by various factors and has been demonstrated both *in vitro *and *in vivo *[35]. A group of inflammatory cytokines including TNF-α, IFN-γ and TGF-β have been suggested to initiate apoptosis [36-40]. In order to determine whether IL-6 is involved in the regulation of apoptosis, we stained cells with TO-PRO-3 dye which can reveal the characteristic morphology associated with apoptosis. TO-PRO-3 is suited as the nuclear staining dyes and an alternative to Hoechst and DAPI, if a UV laser is not available [41,42]. As shown in Figure 6, no typical apoptotic CGCs were observed in either control or IL-6 groups.

### IL-6 stimulated the formation of excitatory synapses

**Figure 7:**
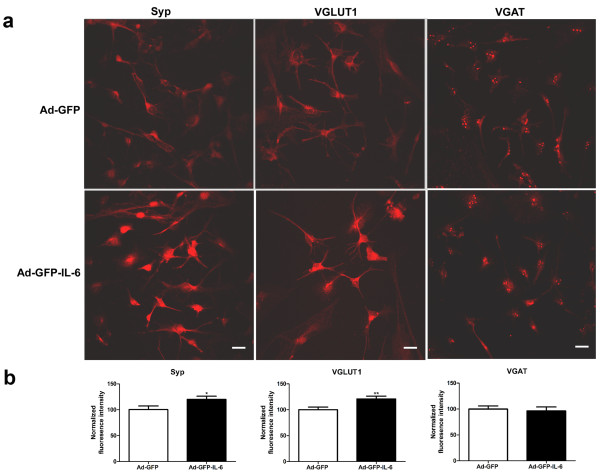
**IL-6 stimulates the formation of excitatory synapses**. (a) Representative photomicrographs of labeling of Syp, VGLUT1 and VGAT in cultured IL-6 and control CGCs, respectively. Scale bars, 25 μm. (b) Quantification of puncta size using automatic image analysis with the NIH ImageJ program for Syp (n = 26 and 25 for control and IL-6 group, respectively), VGLUT1 (n = 23 and 25 for control and IL-6 group, respectively) and VGAT (n = 25 and 24 for control and IL-6 group, respectively). *P < 0.05 and **P < 0.01 compared with control condition.

Autism has been postulated to arise from functional changes in neural circuitry and to be associated with an imbalance between excitatory and inhibitory synaptic transmission, but the mechanisms involved are unknown [43]. To investigate whether an elevation of IL-6 could have an effect on the proportion of excitatory or inhibitory synapses, we examined the synapses formed in IL-6 and control CGCs by using antibodies to synaptic vesicle proteins. An antibody to synaptophysin, a general marker of all synapses, as well as antibodies to VGLUT1 (vesicular glutamate transporter), a marker of excitatory synapses, and to VGAT (the vesicular GABA transporter), a marker of inhibitory synapses, were used in this study (Figure 7). We observed a dramatic increase in the intensity of Synaptophysin and VGLUT1 in IL-6 infected CGCs as compared to control cells (20% and 21% increase, respectively, Figure 7). In contrast, the intensity of VGAT was not altered (Figure 7). These data suggest that IL-6 stimulates the formation of excitatory synapses, while having little effect on inhibitory synapses.

## Discussion

Localized CNS inflammation has been implicated in the development of autism. Cytokines including TNF-α, IFN-γ, IL-1β and IL-12 have been reported to be elevated in the blood mononuclear cells, serum and plasma of autistic subjects [6,20-24]. Recent studies from our laboratory and others have demonstrated that IL-6, IL-8, TNFα, TGF-β1 and MCP-1 are increased in frontal cortex, as well as in the cerebrospinal fluid of autistic subjects [5,7,44]. In this study we used immunohistochemistry to show that IL-6 is significantly increased in the cerebellum of autistic brain as compared with age and sex-matched normal controls. Previously, a single study [7] examined the expression level of IL-6 in the cerebellum of 7 autistic subjects and 7 controls using ELISA. The results from that study demonstrated that the mean value of IL-6 was increased approximately 5 fold in the autistic cerebellum as compared with controls. However due to a large variance in the sample, this observation did not reach significance at the 0.05 level. One possible source for the size of the variance is the selection criteria for the sample. Our results are based on a sample with strictly age- and sex-matched controls, and thus may be more reliable. The results presented here contribute to the growing body of evidence that localized CNS inflammation may contribute to the development of autism.

An elevated cytokine response is associated with autism and IL-6 has been repeatedly found to be increased in the autistic brain. In this study, we developed a neural cell model of IL-6 over-expression with an adenoviral expression vector approach and examined the effects of excessive IL-6 on neural cell properties and functions. We showed that IL-6 over-expression in cultured mouse cerebellar granule and microglial cells significantly impaired neural cell migration and adhesion. Neuronal migration is a fundamental process that determines the final location of neurons in the nervous system, and thus establishes the basis for the subsequent wiring of neural circuits. From cell polarization to target identification, neuronal migration enables neuronal precursors to move across the brain to reach their final destination by integrating multiple cellular and molecular events [33]. Thus, we suggest that increased IL-6 expression in the autistic brain could affect migration and result in altered wiring of neural circuits. Cell migration requires the dynamic regulation of adhesion complexes between migrating cells and the surrounding extracellular matrix [33]. In many cell types, this process involves integrin-mediated adhesion [45-47]. Recently, another study from our laboratory detected a compromised Integrin β1/FAK/Src signaling in autistic lymphoblasts and a significantly decreased migration of autistic lymphoblasts [48]. Whether the inhibitory effect of IL-6 on granule cells migration and adhesion involves the mediation of Integrin β1/FAK/Src signaling pathway remains to be further investigated.

Emerging evidence points to an association of apoptosis with certain neuropsychiatric disorders including autism. Araghi-Niknam and Fatemi [49] demonstrated altered levels of anti-apoptotic Bcl-2 and pro-apoptotic p53 proteins in the parietal cortex of autistic subjects. We also found that the Bcl-2 protein level is decreased and the BDNF-Akt-Bcl-2 anti-apoptosis pathway is down-regulated in the frontal cortex of autistic subjects [10]. In addition, several studies have shown that apoptosis can be initiated by activation of a group of inflammatory cytokines, including TNF-α, IFN-γ and TGF-β [36-40]. Thus, we examined whether IL-6 over-expression promotes neural apoptosis. Our results showed that IL-6 over-expression did not significantly enhance apoptotic changes in the cultured granule cells. It is not clear whether IL-6 is involved in the regulation of apoptosis-related protein Bcl-2 and p53. However, our findings imply that the increased IL-6 in autistic brain may not be responsible for the possible apoptotic changes associated with autism.

Synapses are asymmetric intercellular junctions that mediate neuronal communication. The number, type, and connectivity patterns of synapses determine the formation, maintenance, and function of neural circuitries. Improper synapse formation and function may cause neurodevelopmental disorders, such as mental retardation and autism [50,51], and likely play a role in neurodegenerative disorders, such as Alzheimer's disease [52]. The complexity and specificity of synaptogenesis relies upon modulation of cell adhesion molecues (CAM), that regulate contact initiation, synapse formation, maturation, and plasticity. Disruption of adhesion may result in structural and functional imbalance in synapses. Surprisingly our *in vitro *studies showed that increased IL-6 stimulates granule cell synapse formation, particularly enhanced excitatory synapse formation, while it had little effect on inhibitory synapses. We also demonstrated that IL-6 over-expression in granule cells affected granule cell adhesion and migration, which suggests that IL-6 could be involved in the regulation of CAMs that critically modulate excitatory synaptic formation. It will be important to further study the underlying mechanisms by which IL-6 affects the function of CAMs and the way CAMs may be regulated by IL-6. A number of studies have demonstrated that mutations in the synaptic adhesion molecules neurexin 1 and neuroligins 3 and 4 are associated with autism [51,53]. The scaffolding molecule and interacting protein of neuroligin SHANK3 has also been reported to be altered in some autistic subjects [54,55]. These observations imply an imbalance of neuronal circuitries in autism, which could contribute to the development of the disorder. Our findings, showing that IL-6 is increased in the autistic brain and stimulates excitatory synaptic formation in vitro, provide further evidence to support this hypothesis.

## Conclusions

In conclusion, our study demonstrates that IL-6 was significantly increased in the cerebellum of autistic subjects as compared with age- and sex-matched controls. We also showed that IL-6 over-expression in cerebellar granule cells *in vitro *impaired granule cell adhesion and migration. In addition, we found that IL-6 over-expression stimulated the formation of excitatory synapses of granule cells, while having no effect on the inhibitory synapses. These findings suggest that the elevated IL-6 in the autistic brain could cause an imbalance of neuronal circuits through its effects on neural cell adhesion/migration and synapse formation, and contribute to the development of autism.

## Conflict of interests

The authors declare that they have no competing interests.

## Authors' contributions

XL participated in the design of the study, evaluation and analyses of the results, and drafting of the manuscript. HW participated in conducting most of the experiments, statistical analyses of the results. HZ, AS and CD participated in Western blot and cell culture. MM and WTB participated in results analysis and manuscript edition. All authors read and approved the final manuscript.

## Supplementary Material

Additional file 1 **Table S1**. Study subject information. Click here for file
