# Supplementary material for: IL-6 is increased in the cerebellum of autistic brain and alters neural cell adhesion, migration and synaptic formation
Source: J Neuroinflammation. 2011 May 19;8:52. doi: 10.1186/1742-2094-8-52 (PMC3114764; doi:10.1186/1742-2094-8-52)
Supplement: Additional file 1 — Table S1. Study subject information. [file 1742-2094-8-52-S1.PDF]

Table S1. Study subject information

Case	Age	Sex	Group	PMI(h)	Seizure	Retardation	Medication	Cause of death
1	7	M	Control	12	-	-	Concerta, Clonidone	Drowning
2	8	M	Control	36	-	-	-	Drowning
3	4	F	Control	21	-	-	-	Lymphocytic myocarditis
4	9	F	Control	20	-	-	Albuterol, Zirtec	Asthma
5	6	M	Control	18	-	-	-	Multiple injuries
6	14	M	Control	16	-	-	-	Cardiac Arrhythmia
7	7	M	Autism	20	-	-	-	Drowning
8	8	M	Autism	16	-	-	-	Drowning
9	4	F	Autism	13	-	-	-	Multiple injuries
10	9	F	Autism	24	-	-	-	Smoke inhalation
11	8	M	Autism	12	-	+	-	Drowning
12	14	M	Autism	12	+	+	-	Drowning
